# Importance of a Healthy Tongue: Could It Be a Reflection of Overall Health in Children?

**DOI:** 10.7759/cureus.85574

**Published:** 2025-06-08

**Authors:** Bharat Ram Chowdry Guttikonda, Sandhya J Kadam, Krishna Veni Guttikonda

**Affiliations:** 1 Dentistry, Family Health Care Network, Visalia, USA; 2 Pediatrics, Family Health Care Network, Visalia, USA

**Keywords:** atrophic glossitis, flossing, geographic tongue, halitosis, healthy tongue, macroglossia, oral thrush, tongue hygiene, tongue scraper

## Abstract

This review aims to study the importance of a healthy tongue along with the care of the teeth to maintain the overall good health of the oral cavity. Oral health is often considered secondary to overall health, and tongue hygiene is typically prioritized after tooth care. Poor tongue hygiene leads to halitosis, dry mouth, cavities, altered taste perception, and oral infections. Scraping the tongue while brushing the teeth using a tongue scraper is recommended, but people do not follow it. Checking the tongue is essential to screening in medical, pediatrics, and dental exams. Any changes in color, shape, size, and texture of the tongue may indicate underlying local or systemic diseases. A healthy tongue is pink with healthy papillae, which helps us enjoy the taste of the food we eat. A simple step of taking an extra few seconds for tongue scraping during daily teeth brushing can help to maintain good tongue health.

To write this review, articles from electronic databases such as PubMed/Medline, Google Scholar, and Semantic Scholar are studied. Results found the importance of normal tongue color, texture, shape, and size. The results further found the various tongue pathologies, challenges of maintaining tongue hygiene, and possible future directions to deal with them.

This article explores the importance of tongue hygiene in children and adults by comparing their oral care needs and practices. It also provides an overview of different tongue pathologies based on their color, shape, size, and texture.

## Introduction and background

The tongue is a vital oral organ, and looking at it during a general physical examination can help describe overall health. The tongue is multifunctional. It aids in chewing, swallowing, speaking, and tasting. It is covered by thousands of small, raised bumps known as papillae, which house taste buds. Oral hygiene is important for maintaining good health and preventing oral issues. It is important to develop good dental habits during childhood, like brushing twice daily, flossing regularly, and tongue cleaning to ensure long-term good oral cavity health [1].

Mosaico et al. reported that the tongue carries a load of microorganisms found more frequently in children younger than 18 months, and they studied whether the removal of bacteria on the tongue can help with caries prevention [1]. The results suggested that a combination of a nutritious diet, healthy lifestyles, and specific tongue hygiene protocols can help improve oral health [1].

Poor tongue hygiene causes food particles and bacteria trapped on the dorsum of the tongue in papillae, which can exacerbate conditions such as dry mouth and may contribute to streptococcal infections, tonsilloliths, bad breath, and bad taste in the mouth, affecting the function of different types of papillae. The dorsum of the tongue is particularly prone to debris collection, leading to bad breath, a condition known as halitosis. An unscraped tongue accumulates pathogens, worsening preexisting conditions such as Sjögren’s syndrome and burning mouth syndrome. A dry mouth reduces salivary flow, which can contribute to dental caries and gingival inflammation. Zhang et al. studied tongue microbiota variation and its relation with dental caries in school children [2]. The study suggested that a shift in the tongue microorganisms accompanied dental caries; therefore, the maintenance of a healthy tongue environment may help in the prevention of dental caries [2].

Tongue hygiene is a basic human function, but is often neglected. Pediatricians and dentists can address some of the different demands and applications of tongue hygiene in children and adults to help keep everyone's tongues healthy and ensure overall good health.

## Review

Methodology

For this review, we searched the electronic PubMed/Medline, Google Scholar, and Semantic Scholar databases for peer-reviewed articles that addressed the importance of a healthy tongue, preferably published from 2008 to 2024. All three authors performed an independent search in the mentioned databases. The keywords used were “Flossing, Halitosis, Healthy tongue, Macroglossia, Microglossia, Oral thrush, Tongue hygiene, Tongue Scraper, Teeth brushing”. As a result, a total of 50 articles were identified. The papers were evaluated based on their titles, abstracts, and complete texts, with the primary inclusion criteria being the description of tongue hygiene and the importance of a healthy tongue. The most important exclusion criterion was that the article addressed diseases other than healthy tongue and tongue hygiene. The papers that were not written in the English language were also excluded. The aim was to synthesize the existing evidence and offer critical analysis and insights to provide the information to the providers. The review included 19 articles after the final evaluation.

Discussion

The tongue is a mobile part of the oral cavity, reaching all other parts of the mouth with its movements. Since it carries most of the microorganisms, and at the same time, it is the most mobile part of the oral cavity, the health of the oral cavity largely depends on the condition of the tongue. Limited tongue movements, an unestablished oral health routine, and exposure to external objects such as pacifiers and bottle nipples can increase the chances of oral candidal infection on the tongue in younger children. On examination, a thick white coating on the tongue is present. It can also be seen in breastfed babies. White patches over the tongue spread all over the oral mucosa, leading to possible poor oral intake. Thrush over the tongue in breastfed babies draws attention to the mother's breast hygiene or any underlying systemic illnesses like diabetes. Vainionpää et al. studied 32 infants with oral thrush. In the study, thrush was found to be associated with maternal factors such as mastitis and antibiotic use [3].

An unclean tongue can affect the ability to taste. A neglected tongue can develop a white or yellowish coating and discoloration from bacteria, dead cells, and food particles. Unscraped tongue can contribute to more serious oral gum diseases, cavities, and systemic diseases. Research has shown that bacteria from the mouth, including those on the tongue, can enter the bloodstream, leading to serious heart disease. Janket et al. studied the hypothesis that oral hygiene self-care (OHS) can affect cardiovascular (CVD) mortality and the effects of the use of mouthwash [4]. The study found that taking good care of oral hygiene lowered the risk of CVD mortality as compared to poor oral self-care. Mouthwash use did not show any effects on CVD mortality [4].

Hopkins et al. studied how oral health, including more than just teeth, can affect heart health [5]. Chronic inflammation of the oral cavity involving the teeth and other oral soft tissues can lead to cardiovascular disease, along with endocarditis, diabetes, hypertension, and hyperlipidemia [5].

In another study, Tribble et al. discussed the role of tongue hygiene and its relationship with resting systolic blood pressure [6]. The study suggested that maintaining a healthy tongue microbiome with regular cleaning and adequate dietary nitrate intake can improve resting systolic blood pressure [6].

Importance of Normal Tongue Color, Texture, Shape, and Size

The tongue could be considered a mirror of the patient's overall well-being, as examining the tongue can reveal many things to health professionals. A healthy tongue is usually pink, with a light to dark shade, moist with a thin white coating, and has many papillae, which are tiny bumps that give the tongue a rough texture. It indicates blood circulation and mucous membrane function. Li Q et al. studied tongue color analysis by using hyperspectral images to find the color distribution on the tongue surface to use for tongue disease diagnosis [7]. This approach to color analysis of the tongue is better than the traditional method of achieving meaningful areas of substances and coatings of the tongue [7].

A dry tongue can indicate dehydration. The appearance of the tongue can be based on what the patient eats and drinks. Colorful drinks and candies can leave a distinctive residue on the tongue. Temporary discoloration of the tongue can be related to foods. Specific tongue colors may indicate underlying health conditions. Infection may turn the tongue yellow. A thick tongue coating with a gray-black color could indicate poor intestinal health or digestive issues. Central cyanosis may lead to bluish discoloration of the tongue. Glossitis, the inflammation of the tongue, maybe a component of stomatitis resulting from nutritional deficiency and the use of antibiotics. The tongue can be of normal size, bigger, or smaller. Macroglossia, which is a bigger tongue than the normal size, may indicate underlying congenital conditions and components of known congenital syndromes such as acromegaly, Beckwith-Wiedeman syndrome, and hypothyroidism. An enlarged tongue may also be associated with oral cavity and tongue cancers, trauma, and inflammatory conditions, which more commonly occur in adult patients. The smaller tongue size, microglossia, may be related to nutritional deficiency and starvation [8].

A healthy tongue is free from cracks and sores, has a slightly rough top, and has a shiny, smooth bottom. Hairy tongue occurs when dead skin cells accumulate on the tongue's surface. It is usually harmless. However, the tongue may lose its bumpy texture and become smooth in a condition called atrophic glossitis, which can signify nutritional deficiencies and systemic illnesses. Li H et al. suggested studying the oral microbiome in patients to explore the reason and mechanism of atrophic glossitis [9]. It was found that there was a lower heterogeneity of microorganisms in atrophic glossitis patients than in healthy individuals. The data suggested that *Lactobacillus* and *Saccharomycetales* organisms were associated with the causation of atrophic glossitis [9].

**Figure 1:**
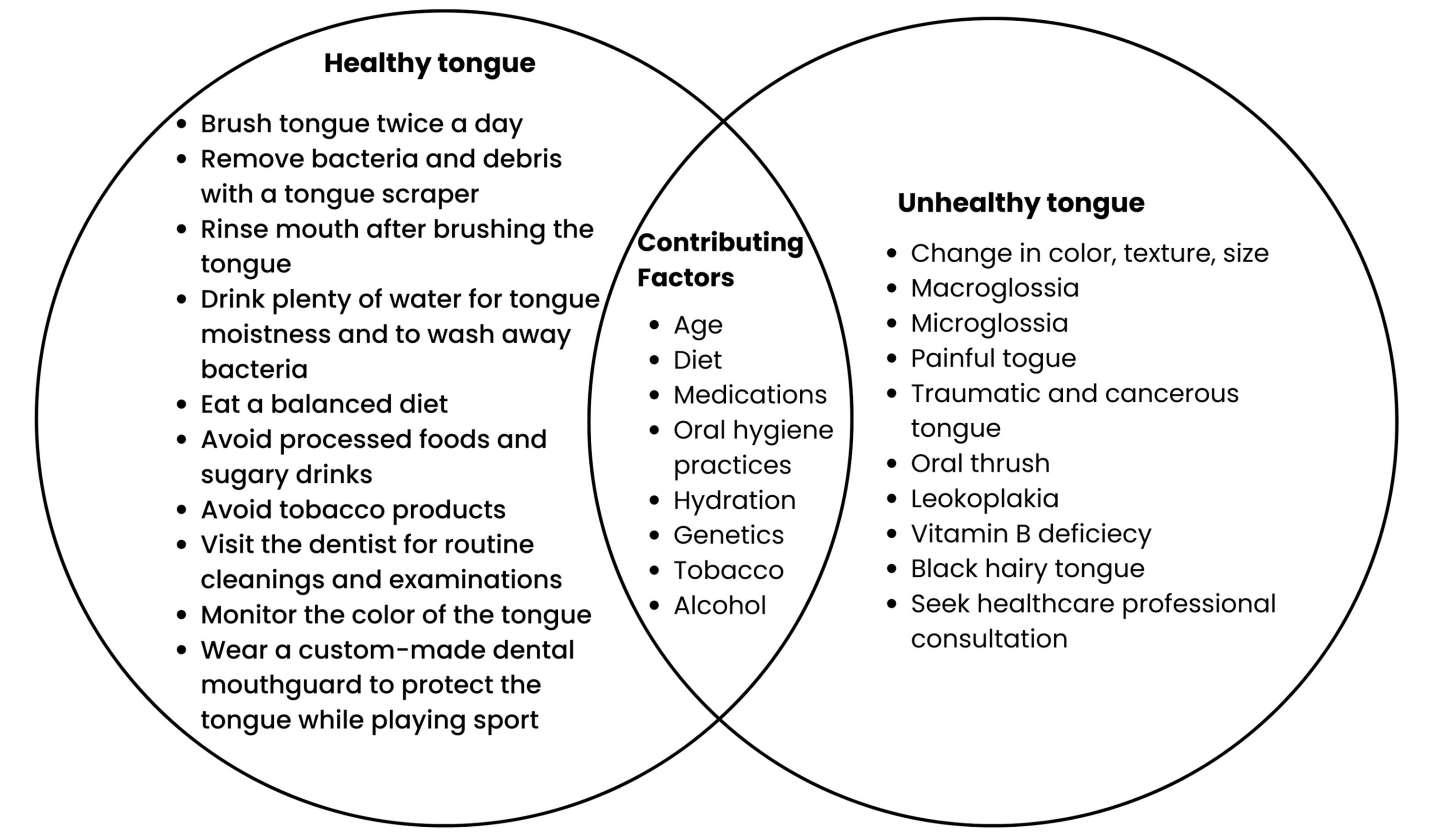
Summary of tongue hygiene [7-10] Figure created by the authors

A normal tongue is symmetrical and round, and it is not too thick or too thin. Geographic tongue is a common, usually benign condition that causes smooth, red patches surrounded by a line like a map that may change in size, shape, and location. The patches usually appear on the top or sides of the tongue, but can also affect the lips or inner cheeks. Geographic tongue is usually painless, but some people may experience a burning sensation or sensitivity to certain foods. The patches may last for days, weeks, or months and then disappear. Geographic tongue is unrelated to infection or cancer, but its association with psoriasis is discussed. Patients are advised to see the doctor if the geographic tongue is persistent or becomes symptomatic. Supportive treatment for the geographic tongue symptoms is advised; since it is correlated to nutritional deficiencies, supplementation of multivitamins and minerals is advised [10]. Please refer to Figure 1 for a summary of tongue hygiene [7-10].

Developmental Considerations in Children

Deciduous teeth form the foundation of permanent teeth. So, it is important to educate and help children care for the oral cavity as a whole organ from early life. The tongue is an active part of their early development in infants and toddlers. Babies use their tongues for feeding and exploring their environment. While most parents focus on cleaning their baby's gums and teeth, the tongue is often neglected. In infants, it is essential to wipe the tongue with a soft, clean, damp cloth after each feeding to remove milk residue that can promote the growth of bacteria and the development of oral thrush. Tongue scraping should aim to remove the soft coating that can accumulate gently over time. This is especially important for bottle-fed babies, as sugary formula milk residue left on the tongue can lead to easy bacterial buildup due to the calorigenic environment [11].

Brecher and Lewis reviewed the eruption of teeth, normal development, and congenital and acquired conditions affecting the mouth [11]. The article suggested the importance of reinforcing positive oral habits even in infancy, with very few teeth, which can help with remineralization of the teeth and establish a lifelong healthy practice. The review also advises paying attention to negative habits of bottle propping and juice intake, as they can promote caries and obesity [11].

As children grow older and develop their teeth, their oral health becomes more complex. Young children who struggle with brushing their teeth properly often overlook their tongues. Children can begin using a toothbrush designed for their age group, with a small head and soft bristles. Children may be able to brush their tongues on their own with guidance by the age of three or four. Parents are encouraged to supervise their children’s brushing habits daily for younger children and at least intermittently for older children at least once a week. At this stage, a tongue scraper may also be introduced to the children. The child must understand how to use it correctly to avoid injury. The tongue should be gently brushed from the back to the front to prevent gagging and remove any buildup. Yu et al. studied the effectiveness of a family-centered approach to improve oral health in children [12]. The study showed that the children had better oral health conditions with fewer dental plaques, white lesions, and cavities whose parents received education and counseling for oral health promotion [12].

Encouragement of other recommendations to look after the oral cavity, such as brushing teeth at least twice a day, flossing teeth each day, using an antibacterial mouthwash once a day, drinking plenty of water to stay hydrated, and going to the dentist regularly for cleanings and exams should be continued by pediatricians during each physical exam visits. Parents can make oral hygiene, including teeth and tongue cleaning, a fun activity. They can be advised to make this activity attractive and rewarding, which may encourage children. Oral health hygiene education can also be done at schools with the collaboration of teachers, dentists, and pediatricians. Saccomanno et al. performed a pilot study to encourage acceptance of healthy oral habits at schools [13]. The children were found to be interested in learning while in school. Conducting an educational session about oral health appeared as a good approach to help children get familiar with dental hygiene tools and learn to use them properly [13].

Challenges of Tongue Hygiene in Children

Many children dislike the sensation of brushing or scraping their tongues, which may make them reluctant to accept it as a daily routine. Children are attracted by an advertisement that shows toothpaste and toothbrushes cleaning the teeth, but not the tongue, which may make children focus more on brushing their teeth, neglecting other parts of their mouth, like the tongue and the roof of the mouth. Another challenge is that children who are still developing fine motor skills may be unable to use the brush effectively. As children grow, they should be gradually taught the right tongue-cleaning technique and encouraged to be more independent in their oral hygiene habits. Challenges in oral health affect tongue hygiene as well. Kumar et al.(14) reviewed the oral health challenges in children and women. Available effective interventions are underutilized both at the community and individual levels due to various reasons, including a lack of integration of oral health into the overall healthcare system and programs, community conditions, poverty, and health literacy of families. The review concluded with recommendations to improve oral health by reforming policy and the system to address the challenges of tongue and oral hygiene [14].

Tongue Hygiene in Teenagers and Adults

As teenagers and adults are responsible for maintaining the health of permanent teeth, oral hygiene needs are more advanced. As age increases, the challenges to oral health increase, and the importance of maintaining proper tongue hygiene becomes even more evident. Adults may experience a greater accumulation of bacterial plaque on their tongues due to diet, lifestyle, and medical conditions. So, they must be advised to avoid substances that can expose the tongue to toxins, like cigarettes and vape pens, and not to smoke or use any tobacco products. These recommendations can also be useful for older kids and teenagers. Along with poor oral hygiene, there are several other reasons for bad breath in adults, such as poor diet, medications, underlying health conditions affecting the immune system, diabetes, and gastrointestinal issues. Kumbargere Nagraj et al.(15) performed a review to study tongue scraping and mouth rinses for halitosis. They found low-to-very-low-certainty evidence, as compared to placebo, that these interventions are effective [15].

The natural sense of taste gets altered in older age as taste buds decrease. Decreased taste sense can make older patients less aware of the oral environment, which may worsen tongue hygiene. In such cases, it is recommended that older adults be educated to use proactive strategies to ensure proper tongue hygiene. Good oral hygiene, a balanced diet, staying hydrated, exercising, and brushing teeth with scraping the tongue may help children and adults. Madiloggovit et al.(16) performed a pilot study to study the effect of tongue brushing on taste perception. The study suggested that brushing the tongue could improve various taste sensations. The study recommended daily tongue brushing as routine personal care for older adults [16].

Comparison of Tongue Hygiene Between Children and Adults

While children and adults need to maintain good tongue hygiene, some key differences exist in their oral care practices and needs. Tongue hygiene requirement for children changes as per age. Infants require gentle cleaning after feedings, while toddlers and young children may need assistance and encouragement to clean their tongues. In contrast, adults generally have the skills and independence to care for their tongues but may face challenges due to lifestyle factors, health conditions, and age. Rickenbacher et al. studied the use of tongue vacuum cleaners, which were accepted by children during professional prophylaxis [17]. They found that it may help promote regular tongue cleaning at home [17].

Sensory issues and unfamiliarity with the practice of cleaning the tongue may lead to resistance in children. In contrast, adults are often more accustomed to maintaining oral hygiene but may neglect the tongue or overlook its importance. For children, oral issues may be simple, but adults may deal with more complex oral health problems, which are often exacerbated by poor tongue hygiene [18].

**Figure 2:**
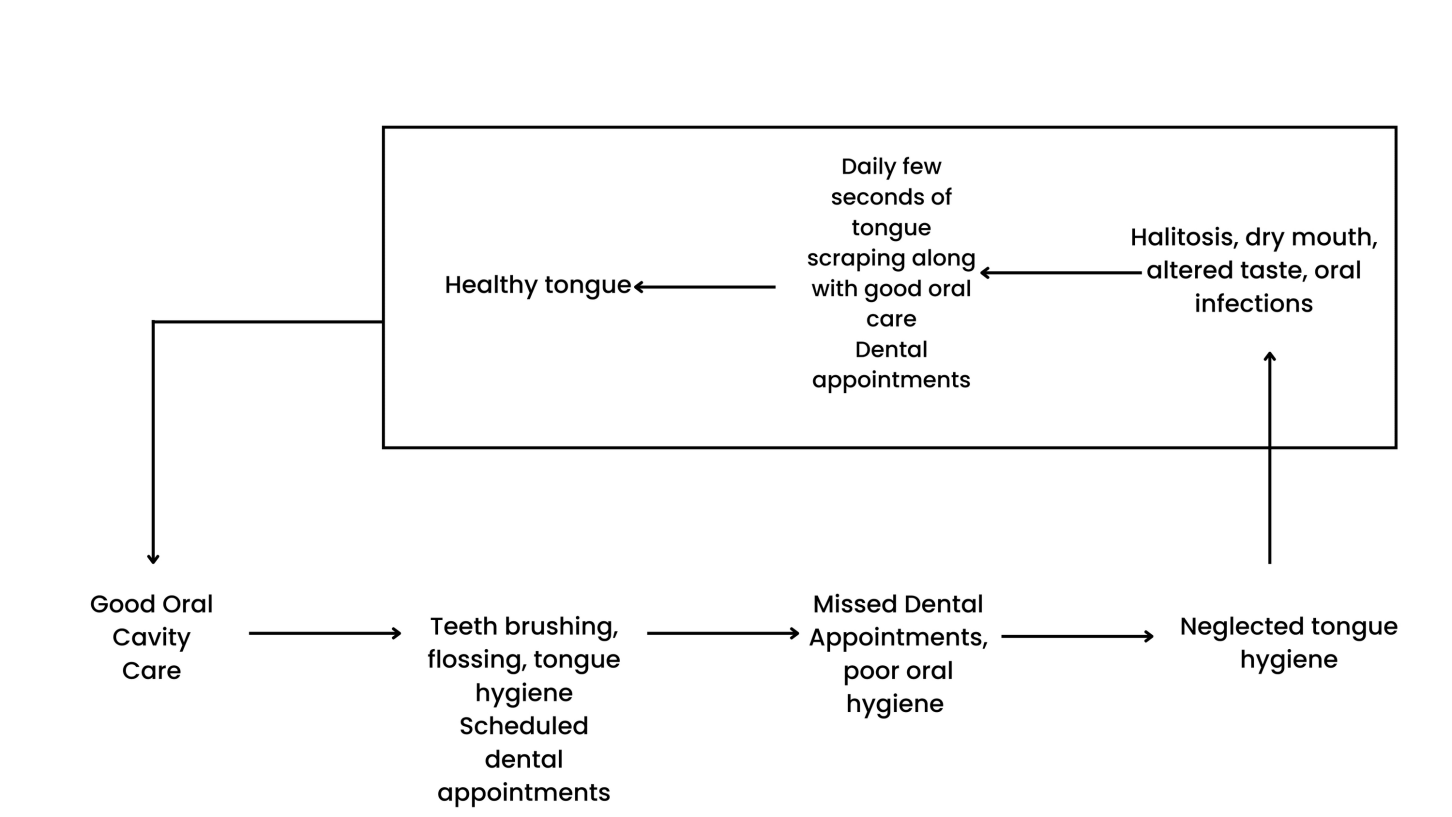
Graphic abstract of the importance of a healthy tongue Figure created by the authors

Tongue scrapers or toothbrushes with tongue-cleaning features may be useful for everyone. However, children require age-appropriate tools and guidance, while adults may have a broader range of options, including specialized mouthwashes or more advanced cleaning devices [19]. Please refer to Table 1 for a comparison of tongue hygiene between children and adults [18,19]. Please refer to Figure 2 for the graphic abstract.

**Table 1:** Comparison of tongue hygiene between children and adults

Factors compared	Children	Adults
Health education	Limited understanding of tongue hygiene	More aware but may need reminders
Role of parents	May need parental guidance and supervision; parental education and encouragement are the key factors	Self-independent self-motivation and individual habits are the keys
Use of cleaning tools	Age-wise use of cleaning tools, from soft cloths to soft toothbrushes; gentle cleaning with special care to avoid injury	Regular tools may be enough; fewer chances of injury during cleaning
Risk of tongue infections	High risk of tongue infections due to failure to use the proper technique	High risk of oral infections due to an underlying medical condition
Challenges of tongue hygiene	Resistance sensitivity of the tongue; inability to reach the back of the tongue	Lifestyle, diet, medical conditions

Recommendations

The collaboration of dentists and primary care physicians (PCPs), including pediatricians, may become more effective in reinforcing the importance of tongue hygiene and maintenance of a healthy tongue.

Along with twice daily teeth brushing, the message of daily tongue scraping can be given during routine well-child check examinations to help maintain good overall oral cavity health and prevent oral cavity issues.

Further research for the education, learning, and training of PCPs may have an impressive impact on the oral health of children. Accessibility to timely referral to pediatric dentists can help deal with pathological conditions of the tongue [17].

## Conclusions

Good oral hygiene habits in childhood, including a few seconds of daily tongue cleaning during toothbrushing, can lay the foundation for lifelong oral health. Educating and motivating parents and children early and further research to train healthcare professionals, can support consistent tongue hygiene practices across all age groups.
